# Polymeric LabChip Real-Time PCR as a Point-of-Care-Potential Diagnostic Tool for Rapid Detection of Influenza A/H1N1 Virus in Human Clinical Specimens

**DOI:** 10.1371/journal.pone.0053325

**Published:** 2012-12-28

**Authors:** Hyun-Ok Song, Je-Hyoung Kim, Ho-Sun Ryu, Dong-Hoon Lee, Sun-Jin Kim, Deog-Joong Kim, In Bum Suh, Du Young Choi, Kwang-Ho In, Sung-Woo Kim, Hyun Park

**Affiliations:** 1 Zoonosis Research Center, Department of Infection Biology, Wonkwang University School of Medicine, Iksan, Jeonbuk, Republic of Korea; 2 NanoBioSys Inc., Geumcheon-gu, Seoul, Republic of Korea; 3 Department of Laboratory Medicine, Kangwon National University School of Medicine, Chuncheon, Kangwon-do, Republic of Korea; 4 Department of Pediatrics, Wonkwang University School of Medicine, Iksan, Jeonbuk, Republic of Korea; 5 Department of Medicine, Korea University School of Medicine, Seoul, Republic of Korea; Michigan State University, United States of America

## Abstract

It is clinically important to be able to detect influenza A/H1N1 virus using a fast, portable, and accurate system that has high specificity and sensitivity. To achieve this goal, it is necessary to develop a highly specific primer set that recognizes only influenza A viral genes and a rapid real-time PCR system that can detect even a single copy of the viral gene. In this study, we developed and validated a novel fluidic chip-type real-time PCR (LabChip real-time PCR) system that is sensitive and specific for the detection of influenza A/H1N1, including the pandemic influenza strain A/H1N1 of 2009. This LabChip real-time PCR system has several remarkable features: (1) It allows rapid quantitative analysis, requiring only 15 min to perform 30 cycles of real-time PCR. (2) It is portable, with a weight of only 5.5 kg. (3) The reaction cost is low, since it uses disposable plastic chips. (4) Its high efficiency is equivalent to that of commercially available tube-type real-time PCR systems. The developed disposable LabChip is an economic, heat-transferable, light-transparent, and easy-to-fabricate polymeric chip compared to conventional silicon- or glass-based labchip. In addition, our LabChip has large surface-to-volume ratios in micro channels that are required for overcoming time consumed for temperature control during real-time PCR. The efficiency of the LabChip real-time PCR system was confirmed using novel primer sets specifically targeted to the hemagglutinin (HA) gene of influenza A/H1N1 and clinical specimens. Eighty-five human clinical swab samples were tested using the LabChip real-time PCR. The results demonstrated 100% sensitivity and specificity, showing 72 positive and 13 negative cases. These results were identical to those from a tube-type real-time PCR system. This indicates that the novel LabChip real-time PCR may be an ultra-fast, quantitative, point-of-care-potential diagnostic tool for influenza A/H1N1 with a high sensitivity and specificity.

## Introduction

Influenza A virus, including swine influenza and avian influenza, is a highly contagious virus that causes annual epidemic and sometimes pandemic disease in humans [1]. Thus, it is extremely important to diagnose influenza A infection using a rapid, sensitive, and specific method in order to prevent outbreaks [2]–[4]. Current detection methods for human influenza A virus include (1) virus isolation after cell culture, (2) rapid immunochemical assays, such as enzyme-linked immunosorbent assays and immunochromatographic test, and (3) polymerase chain reaction (PCR)-based assays [5],[6]. Rapid diagnostic tests (RDT) are simple and easy methods for detecting influenza that require minimal time and cost. Further, they do not require special equipment or expertise. In addition, RDT provides results in 10–30 min. Given these advantages, RDT is the most common diagnostic method for influenza infection. However, the sensitivity of RDT kits ranges from 20–90% for seasonal influenza and 10–70% for the pandemic influenza strain A/H1N1 of 2009 [7]–[15].

Real-time PCR is the most accurate and powerful method and has a high sensitivity [16]. Therefore, many real-time PCR-based assays have been developed for detecting individual subtypes or specific pandemic influenza strains [17]–[23]. Although no real-time PCR assay is 100% sensitive, their sensitivities are considerably higher than that of RDT. However, real-time PCR assays take more time (2–5 h) than RDT, even though automated RNA extraction and PCR machines have significantly reduced the reaction time. Recently, Kawai et al. reported a novel detection method, called the RT-SmartAmp assay, for one-step detection of the pandemic influenza strain A/H1N1 of 2009 [24]. Using this PCR system, the reaction time was reduced to 40 min. The sensitivity of the assay was not satisfactory (54.9%), although it was slightly improved compared to that of current influenza diagnostic tests using lateral flow immuno-chromatography (43.1%). Thus, it is necessary to develop an ultra-fast real-time PCR system for detecting a single copy of an influenza A viral gene by using highly specific primers.

Microfluidic chip technology has been increasingly applied to biological and medical systems [25]–[28]. In particular, fluidic chip-based real-time PCR has received attention for its ability to achieve rapid molecular quantitative analysis of infectious disease pathogens due to its excellent sensitivity and specificity [29]–[32]. In the present study, we developed and validated a fluidic chip-type real-time PCR system. It achieved rapid quantitative analysis (15 min to perform 30 cycles of real-time PCR). In addition, it was portable (5.5 kg in weight) and economical because of disposable plastic chips. Using clinical samples of influenza A/H1N1, we established that the fluidic chip-based real-time PCR system had excellent sensitivity and specificity, which were nearly identical to those of a conventional tube-type real-time PCR system.

## Results and Discussion

### Construction of the LabChip real-time PCR system

In our study, polymer chips were fabricated for such reasons as low cost, light weight, and excellent processing ability. Price determination of disposable diagnostic chip depends heavily on both raw material cost and fabrication cost in industries. The cost of our plastic LabChip is much cheaper than other types of LabChips such as glasses and silicon wafers. Boro-float glasses (e.g. Corning Pyrex), borosilicate glasses (e.g. Schott B270) and photostructurable glasses (e.g. Schott Foturan) are approximately 10–20 cent/cm^2^, 5–15 cents/cm^2^, and 20–40 cents/cm^2^ in raw materials cost [33]. The cost of Si wafer (e.g. LG Siltron) is close to 12–16 cent/cm^2^. However, the cost of polymers like PMMA (Poly-methyl methacrylate) is only about 0.2–2 cent/cm^2^ [33]. In addition, conventional microfluidic chip based on silicone and glass needs multi-step and expensive fabrication process during cleaning, deposition, lithography, etching, and dicing for the generation of complex structure such as channel reaction chamber and detection chamber [34]. However, with polymers, it is possible to generate multilayer structures by one-step injection-molding method.

**Figure 1:**
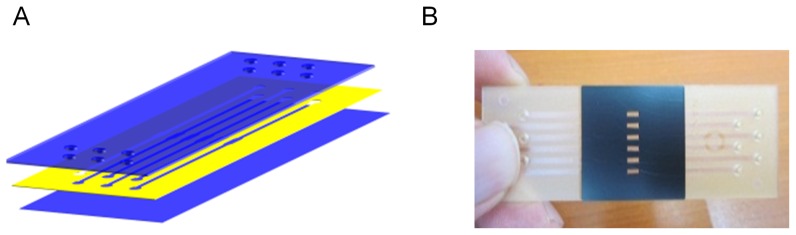
Construction of the plastic LabChip. The schematic diagram (A) and actual image (B) of the fluidic flow chip.

The fluidic flow chip was designed based on a simulation by the COMSOL 3.5 program (COMSOL, Sweden) to make a smooth flow structure of the fluidic channels. Three plastic layers were used to construct the inlet, outlet, fluidic channel, and reaction chambers in a chip measuring 72×25×1.5 mm. The inlet and outlet holes were fabricated to fit a 200-µL pipette tip for convenient sample injection and removal. Its fluidic channel was 1 mm in width and 250 µm in depth, while its reaction chamber was 1 mm in width and 3 mm in length. The reaction volume for the fluidic flow chip was 15 µL for 6 channels (Figure 1).

**Figure 2:**
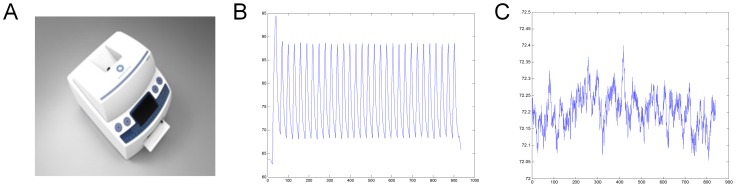
Thirty cycles of the ultra-fast LabChip real-time PCR system. The PCR was completed in 15 min within a 0.5°C temperature variation. A. View of NBS UltraFast LabChip Real-time PCR G2–3. B. Plot of 30 thermocycles over 900 s (15 min). C. Close-up view of 72°C, ranging from 72.08°C to 72.40°C, indicating less than 0.5-degree variation.

The real-time PCR system had 3 major parts, consisting of a thermal cycle control, an optical detector, and data analysis software. A rapid thermal cycler was developed that could perform 30 cycles of real-time PCR in 15 min within 0.5°C temperature variation (Figure 2). The optical detector detected green fluorescence using 490-nm excitation and 530-nm emission filters. Its filtered signal was amplified through a photomultiplier tube. Software developed by NanoBioSys Inc. was installed for user-friendly quantitative analysis of the amplified DNA signals.

### Validation of LabChip real-time PCR

**Figure 3:**
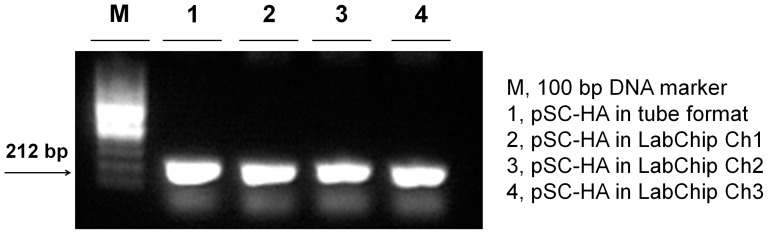
Amplification efficiency in the plastic chip. A positive control cDNA was amplified by tube-type and fluidic chip-type PCR. The electrophoresed amplicons are shown in an agarose gel.

**Figure 4:**
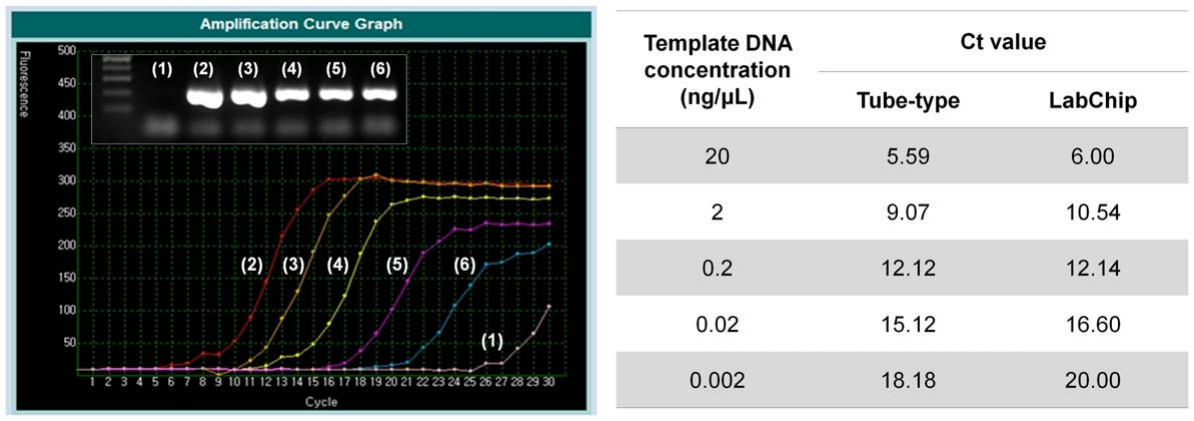
Detection sensitivity of LabChip real-time PCR. LabChip real-time PCR was compared to conventional tube-type real-time PCR for its sensitivity to detect positive template DNA. The Ct values obtained from the LabChip real-time PCR were comparable to those from tube-type real-time PCR (Roche LightCycler 1.5).

**Figure 5:**
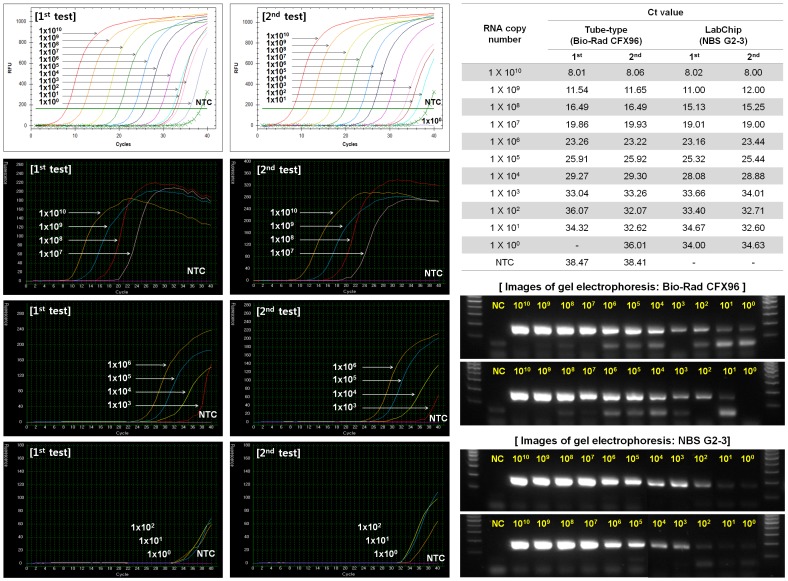
LOD (Limit of detection) of LabChip real-time PCR. Serial dilution of *in vitro* transcribed RNA was tested using LabChip real-time PCR and tube-type real-time PCR (Bio-Rad CFX96). The Ct values obtained from both PCR systems were compared in a table. Test was performed in duplicate. PCR amplicons were confirmed by gel electrophoresis. NTC, no template control.

To determine whether DNA was amplified as efficiently in the designed plastic chip as in a plastic tube, we tested and compared a Bio-Rad real-time PCR system (CFX96) and a NanoBioSys (NBS) UltraFast LabChip real-time PCR system (G2–3) using a positive control plasmid containing Hemagglutinin (HA) cDNA. The amplicons obtained by tube-type and fluidic chip-type PCR were gel electrophoresed. As shown in Figure 3, the amplification efficiency in the plastic chip was not significantly different from that in the tube. Real-time PCR performance was compared using a serial dilution of positive DNA plasmids with a Roche real-time PCR system (LightCycler 1.5) and an NBS UltraFast LabChip real-time PCR system (G2–3). As shown in Figure 4, the cycle threshold (Ct) values obtained from both PCR assays were equivalent. In addition, the limit of detection was further assessed with both PCR assays. Serial dilution of *in vitro* transcribed RNA was tested (Figure 5). Real-time PCR was performed in duplicate to determine the minimum RNA copy number that can be detected. NBS UltraFast LabChip real-time PCR system (G2–3) could detect as few as 1 copy of *in vitro* transcribed RNA with good reproducibility (Figure 5). Similar result was also obtained with tube-type real-time PCR system (Bio-Rad CFX96). However, the linear standard curves were detected from 10^10^ copies to 10^3^ copies per reaction by both PCR assays. At lower RNA concentration (10^2^−10^0^ copies per reaction), primer dimers were observed and thereby the Ct values were not stable. Interestingly, the formation of primer dimers at lower RNA concentration was relatively reduced in LabChip real-time PCR system as shown in electrophoresed gel images. These findings indicated that real-time PCR in the fluidic plastic chip was efficient, and the data were comparable to those obtained in tube-type chambers of other commercial real-time PCR machines.

### Comparative analysis of tube-type and LabChip real-time PCR using clinical specimens

**Figure 6:**
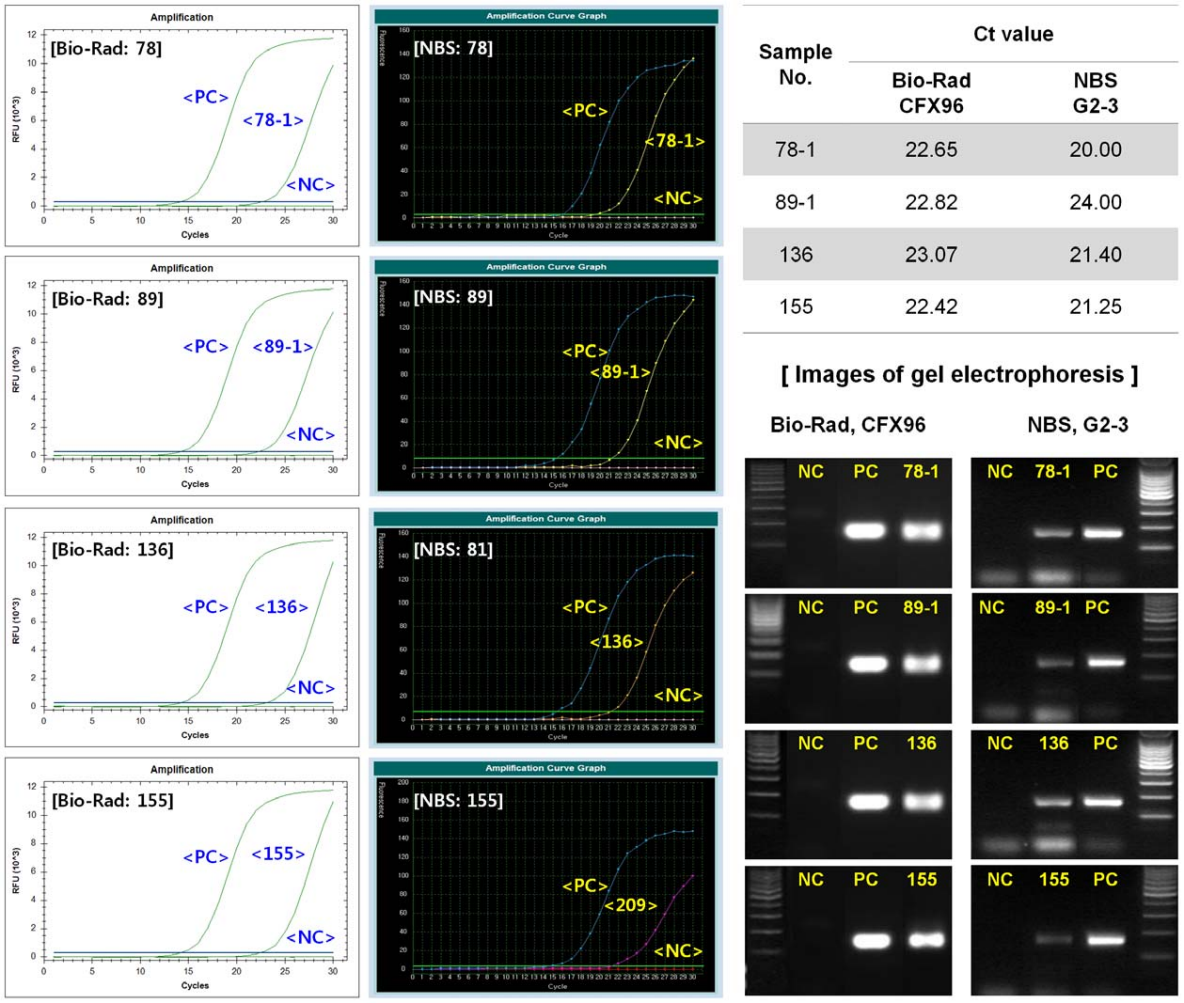
Clinical evaluation of LabChip real-time PCR. Clinical swab samples were used to evaluate the LabChip real-time PCR. The results were compared to those from tube-type real-time PCR (Bio-Rad CFX96). Resultant examples of both real-time PCR assays are shown. PCR amplicons were further analyzed by gel electrophoresis. PC, positive control; NC, no template control.

A clinical evaluation was further conducted to determine whether the plastic fluidic chip-based real-time PCR was suitable for the quantitative analysis of human clinical samples. A total of 85 clinical samples were evaluated, including 72 samples positive for influenza infection and 13 negative samples. The evaluation of clinical samples was conducted by comparing the diagnostic results from our ultra-fast LabChip real-time PCR system (G2–3) to those from tube-type real-time PCR using Bio-Rad CFX96. The results were analyzed by observing the pattern of the amplification curve, Ct values, and the amplified DNA band by agarose gel electrophoresis. Our chip-type real-time PCR assay was able to detect all 72 positive samples with comparable amplification curves and Ct values as those of the tube-type real-time PCR assay; the assay identified 72 samples as positive and 13 as negative, showing 100% sensitivity and specificity (Figure 6 and Table S1). In some cases, lower Ct values were obtained with our LabChip real-time PCR assay, suggesting that our plastic fluidic chip-based real-time PCR system was also valid for testing clinical specimens. The results of the PCR assays on the clinical samples are summarized in Table 1. The evaluation was carried out using 2 independent PCR primer sets, and the same results were obtained with both the tube- and chip-type real-time PCR assays.

**Table 1:** Comparison of tube-type and LabChip real-time PCR for detecting influenza A/H1N1.

	Tube type PCR			LabChip PCR		
Clinical	(Bio-Rad CFX96)			(NanoBioSys G2–3)		
sample	Positive	Negative	Total	Positive	Negative	Total
Negative	0	13	13	0	13	13
Positive	72	0	72	72	0	72
Total	72	13	85	72	13	85

PCR, polymerase chain reaction.

RNA extraction and cDNA synthesis were preceded for both real-time PCR assays.

The evaluation was further performed using other subtypes of influenza A virus strains which can co-circulate in a given influenza season. In particular, we have tested various avian influenza strains isolated from chickens, ducks, and birds. Viral RNA was purified from egg-grown viruses and used for the real-time PCR evaluation. As shown in Figure S1, the LabChip real-time PCR assay exhibited excellent specificity with comparable Ct values to that of tube-type real-time PCR assay. Additional validation was conducted to analyze the cross-reactivity using other related respiratory viral and bacterial pathogens. Evaluation was carried out using clinical samples infected with respiratory viruses such as adenovirus, respiratory syncytial virus and metapneumovirus as well as a bacterium causing tuberculosis. All clinical samples were confirmed for infection with virus and bacterial cultivation. As a result, no cross-reactivity was shown in both tube-type and LabChip real-time PCR assays (Figure S2). Every independent test resulted in similar Ct values from positive DNA plasmid control showing the difference less than 1.

### New paradigm for rapid diagnosis of infectious diseases using LabChip real-time PCR

**Figure 7:**
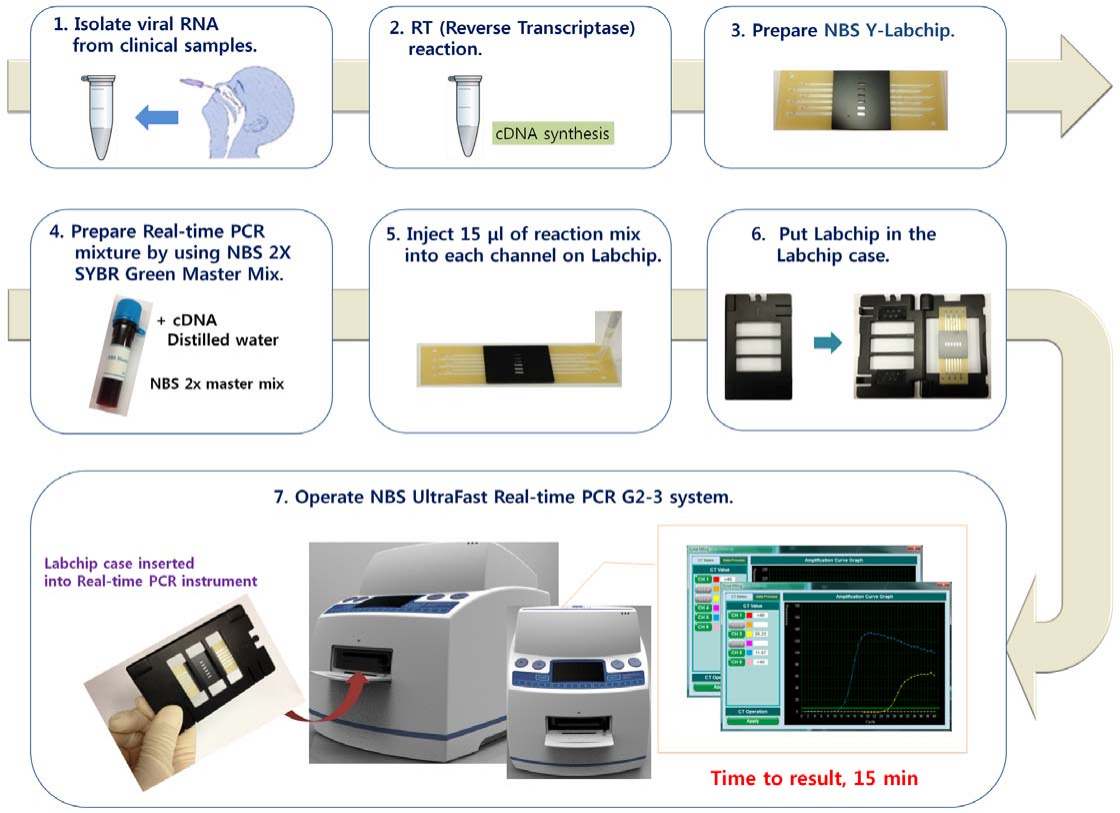
Flowchart of the ultra-fast LapChip real-time PCR to detect influenza A virus. Viral RNA is isolated from clinical samples and reverse transcription is performed to generate cDNA. SYBR green real-time PCR is conducted using synthesized cDNA in a LabChip. Ultra-fast real-time PCR is used, and the results are obtained within 15 min.

Based on our results, we suggest that the ultra-fast LabChip real-time PCR system (G2–3) provides a faster and more accurate system for diagnosing various infectious diseases (Figure 7). In this system, viral RNA was isolated and reverse transcribed to form cDNA, which was subsequently amplified similar to conventional tube-type real-time PCR. Here, disposable plastic chips were used instead of tubes. Importantly, the ultra-fast LabChip real-time PCR system (G2–3) completed 30 cycles of real-time PCR within 15 min. This was approximately 2–4 times faster than conventional tube-type real-time PCR using the Roche system (LightCycler 1.5) or the Bio-Rad system (CFX96). Several types of on-chip PCR have been described for reducing reaction time, system size, and reaction cost. These include flow-through PCR based on silicon and glass substrate chips and droplet-type digital PCR [26],[28]. However, commercial-quality LabChip-based real-time PCR systems that are comparable to conventional real-time PCR systems have not been successfully generated. To overcome technical barriers, we focused on developing a fast thermal cycler with an acceptable minimum temperature variation, reducing the size and weight of the control system by improving the detection method, and utilizing inexpensive disposable fluidic chambers in order to achieve highly efficient real-time PCR comparable to conventional real-time PCR systems. These critical issues were mostly solved by developing polymeric LapChip. Because polymeric LabChip has large surface-to-volume ratios in micro channels that are required for overcoming time consumption to control temperature during real-time PCR.

The advantages of our LabChip real-time PCR system (G2–3) for point-of-care-potential diagnosis include (1) rapid quantitative analysis, which requires only 15 min to perform 30 cycles of real-time PCR, (2) its portable design and small size, weighing only 5.5 kg, and (3) the low reaction cost due to the use of disposable plastic chips. Roche's real-time PCR system uses glass capillary-type reaction chambers with a fast air-blow system in order to perform 30 cycles of real-time PCR in 30–50 min. On the other hand, Bio-Rad's system uses conventional tube-type reaction chambers and a conventional heat block to carry out 30 cycles of real-time PCR in 50–70 min. Our LabChip real-time PCR system uses a renovated plastic Labchip, a miniaturized fast temperature control, and a fluorescence detection system to obtain high efficiency. As such, this system can perform 30 cycles of real-time PCR in 15 min. The LabChip real-time PCR system was twice as fast as Roche's LightCycler 1.5 system (data not shown). This is important, because Roche's LightCycler is one of the fastest real-time PCR systems currently available. Roche's LightCycler is 3–4 times heavier and larger than that developed in the present study. Further, the reaction cost is also greater than that of the LabChip real-time PCR system. Thus, the LabChip-based real-time PCR system can be used for rapid, quantitative detection of infectious diseases.

Taken together, these results demonstrate that the LabChip real-time PCR is a strong candidate for use as a ubiquitous point-of-care-potential test system for the rapid, accurate, quantitative diagnosis of various infectious diseases, such as influenza A/H1N1.

## Materials and Methods

### Primers and synthesized positive-control DNA template

Primers used in the real-time PCR assay were designed based on the sequence of HA obtained from the Influenza Virus Resource, including GenBank accession number GA131023. Primers were designed by Primer3 (v.0.4.0) software with melting temperatures (Tms) of 67–72°C, which are optimal for the NBS SYBR Green I Real-time PCR Master Mix (NanoBioSys Inc., Korea). Two primer sets were used for the real-time PCR assay. The first had the following sequences: 5′- GCT GGA TCC TGG GAA ATC CAG AGT G-3′ (forward primer) and 5′-GTT CGA GTC ATG ATT GGG CCA TGA A-3′ (reverse primer). The other was provided by Dr. H.P. (unpublished data).

The nucleotide sequences amplified by the above primers were synthesized or amplified to generate DNA templates for use as positive controls in the real-time PCR assay. Synthesized nucleotide sequences were cloned into a pUC57 vector with *Bam*HI and *Apa*I restriction enzyme sites (Cosmo Genetech Co. Ltd., Korea). The amplified full sequence of the HA gene segment was cloned into a pSC-A-amp/kan vector (Stratagene, USA). This was performed by RT-PCR as previously described [35]. Briefly, 1 µg of total viral RNA was reverse transcribed using 20 pmol of a gene-specific primer (5′-AGC RAA AGC AGG-3′) at 50°C for 90 min. Then, 30 cycles of PCR were performed using an HA-specific primer set (HA-1: 5′-AGC AAA AGC AGG GGA AAA TA-3′, HA-1778R: 5′-AGT AGA AAC AAG GGT GTT TT-3′).

### *in vitro* transcription of positive-control DNA for the determination of detection limit

The positive DNA template was amplified with designed primers and cloned into pGEM-T Easy vector (Promega, USA). The resultant plasmid DNA was linearized with *Pst*I restriction enzyme and purified with a PCR clean-up kit (Labopass, Cosmo Genetech Co. Ltd., Korea). Purified DNA was transcribed with T7 RNA polymerase using the RiboMAX™ Large Scale RNA Production System (Promega, USA). RNA transcript was further purified with the Total RNA Isolation kit (MACHEREY-NAGEL, Germany) and quantified with spectrophotometer at 260 nm. *in vitro* transcribed RNA was serially diluted and reversed transcribed for the determination of detection limits.

### Ethics

Approval for the use of swab samples for this study was obtained from the Wonkwang University Hospital Institutional Review Board (Approval No. WKUHIRB). Written informed consent was obtained from patients involved in this study, and the study was approved by the ethics committee (director, Eun-Taik Jeong, M.D., Ph.D.).

### Clinical specimens and sample processing

Nasopharyngeal swab specimens of influenza A/H1N1 were obtained from Wonkwang University Hospital. Influenza infections were confirmed using commercially-available PCR assays (Bioneer Inc. Korea and Seegene Inc. Korea). For each swab sample, RNA was extracted with the Total RNA Isolation kit according to the manufacturer's instructions (MACHEREY-NAGEL, Germany). RT-PCR was performed either with the ReverTra Ace qPCR RT kit (Toyobo, Japan) or the SuperScript III First-strand cDNA Synthesis system (Invitrogen™, USA) to obtain cDNA from the extracted RNA.

### Microfluidic LabChip real-time PCR based on SYBR green

Polymeric injection-molded top-side PC plate was fabricated with inlet and outlet. Bottom-side flat PC plate was fabricated using metal mold and injection-molding machine. Both PC plates were attached using adhesive tape to generate microfluidic channels. Total viral RNAs isolated from swap samples were used for the synthesis of cDNA by reverse transcription as manufacturer's protocol (See above, “Sample processing”). Subsequently, real-time PCR reaction was prepared using NBS SYBR Green I Real-Time PCR Master Mix Kit (NanoBioSys Inc., Korea). 10–20% (v/v) of synthesized cDNA and 1 µM of each primer were added, and 15 µL reaction mixture was loaded into each channel of LabChip. LabChip filled with reaction mixture was placed onto the LabChip case and further injected into the ultra-fast LabChip real-time PCR G2–3 system (NanoBioSys Inc., Korea). According to software protocol (pre-denaturation at 95°C for 8 s then 30 cycles at 95°C for 8 s and 72°C for 14 s), real-time PCR was performed. As real-time PCR proceeded, real-time data were plotted on the screen and then sigmoidal curves for amplified pathogen DNAs were appeared. After curve fitting, Ct values and reaction time of real-time PCR were appeared on the screen. It took 15 min for 30 cycles of real-time PCR. Each reaction included the no template control (NTC) to determine whether it was positive or negative. A sample with a Ct value less than 31 was considered positive in cases where the Ct value of the NTC (No Template Control) was not assigned.

### Traditional real-time PCR based on SYBR green

Real-time PCR was performed using the NBS SYBR Green I Real-Time PCR Master Mix Kit (NanoBioSys Inc., Korea). For a reaction volume of 20 µL, 10–20% (v/v) of cDNA and 1 µM of each primer were added. Real-time PCR was carried out using a CFX96 thermocycler (Bio-Rad Laboratories Inc., USA) as follows: pre-denaturation at 95°C for 3 min then 30 cycles at 95°C for 10 s and 72°C for 14 s. Each reaction was conducted in duplicate, including the no template control (NTC) to determine whether it was positive or negative. A sample with a Ct value less than 31 was considered positive in cases where the Ct value of the NTC was not assigned.

## Supporting Information

Figure S1 **Cross-reactivity test against different influenza subtypes.** As listed in Table, various subtypes of influenza virus are tested for analyzing specificity of PCR assays. Ct values from both PCR assays are listed in the table. Test was performed in duplicate and the representative graphs of amplification curve are shown. PC, positive control; NC, no template control.
(TIF)

Figure S2 **Cross-reactivity test against other respiratory viral and bacterial pathogens.** Each box shows the representative graphs of amplification curve from both tube-type and LabChip real-time PCR assays (Test was performed in duplicate). The gel images of PCR results are shown on the rightmost. Only positive control display positive peak is detected from both PCR assays. Adenovirus (n = 9), metapneumovirus (n = 8), Respiratory syncytical virus (n = 5). For tuberculosis (TB) test, genomic DNA of *Mycobacterium tuberculosis* was extracted from patient sputum and used for PCR individually (1) or together with H1N1 positive DNA control (Mixed, 2). Positive signals are shown only from positive control (PC) and mixed sample (2). The difference of Ct values positive control (PC) and mixed sample (2) are less than 1. NC, no template control.
(TIF)

Table S1 **Comparison of Ct values from tube-type and LabChip real-time PCR.**
(DOC)
