# Supplementary figures and images for: Polymeric LabChip Real-Time PCR as a Point-of-Care-Potential Diagnostic Tool for Rapid Detection of Influenza A/H1N1 Virus in Human Clinical Specimens
Source: PLoS One. 2012 Dec 28;7(12):e53325. doi: 10.1371/journal.pone.0053325 (PMC3532060; doi:10.1371/journal.pone.0053325)

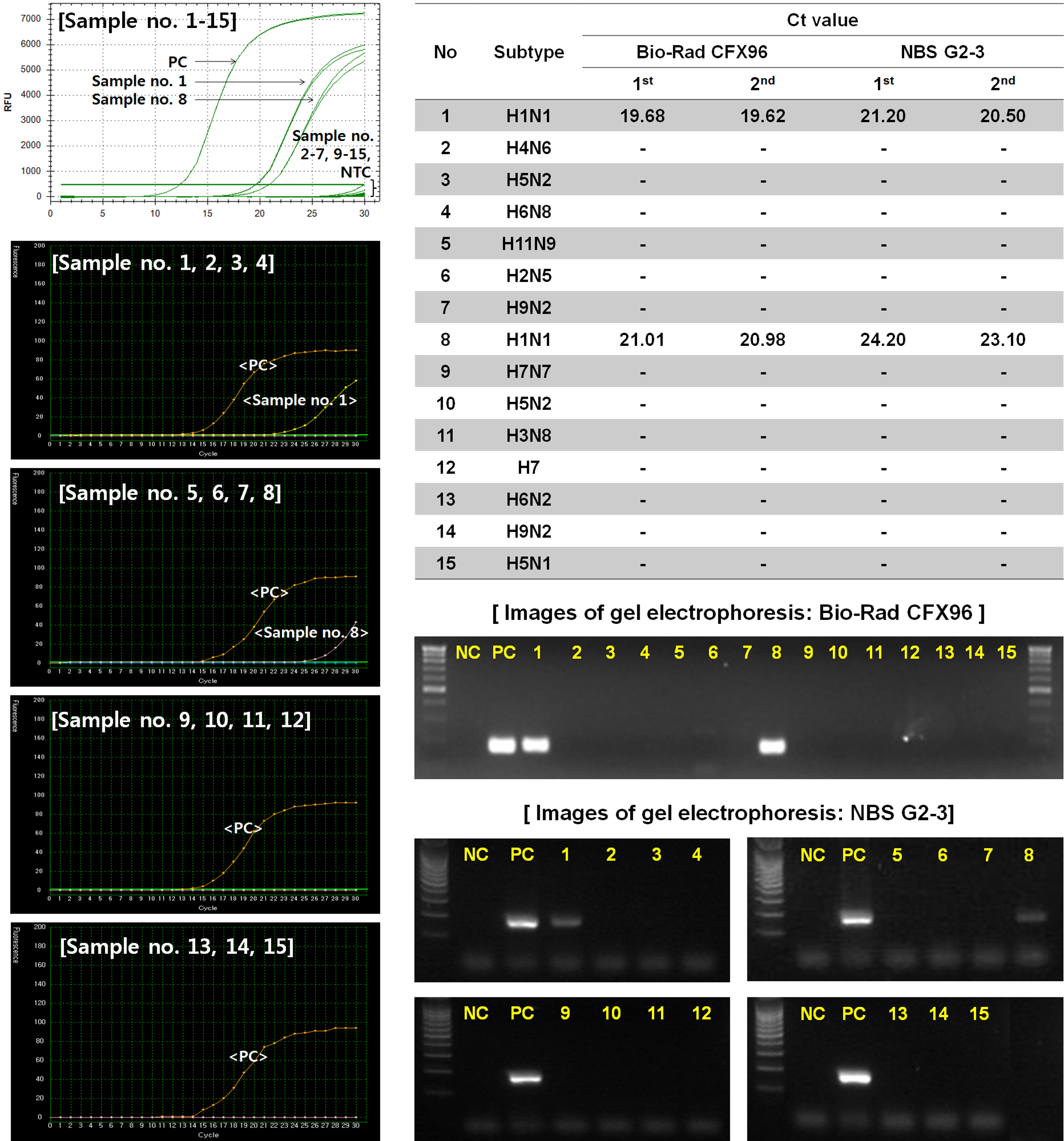

Supplement: Figure S1 — Cross-reactivity test against different influenza subtypes. As listed in Table, various subtypes of influenza virus are tested for analyzing specificity of PCR assays. Ct values from both PCR assays are listed in the table. Test was performed in duplicate and the representative graphs of amplification curve are shown. PC, positive control; NC, no template control. (TIF) [file pone.0053325.s001.tif]

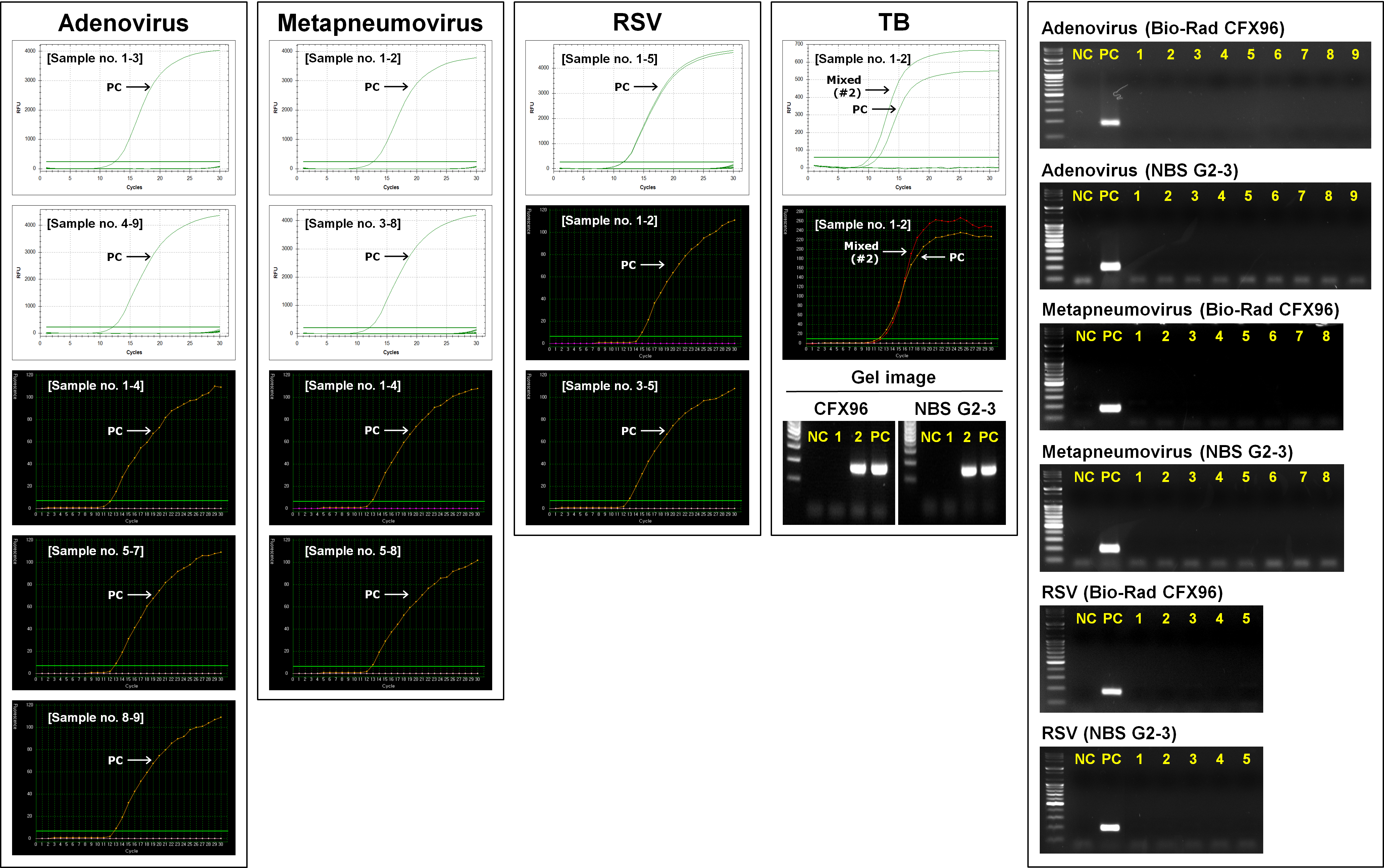

Supplement: Figure S2 — Cross-reactivity test against other respiratory viral and bacterial pathogens. Each box shows the representative graphs of amplification curve from both tube-type and LabChip real-time PCR assays (Test was performed in duplicate). The gel images of PCR results are shown on the rightmost. Only positive control display positive peak is detected from both PCR assays. Adenovirus (n = 9), metapneumovirus (n = 8), Respiratory syncytical virus (n = 5). For tuberculosis (TB) test, genomic DNA of Mycobacterium tuberculosis was extracted from patient sputum and used for PCR individually (1) or together with H1N1 positive DNA control (Mixed, 2). Positive signals are shown only from positive control (PC) and mixed sample (2). The difference of Ct values positive control (PC) and mixed sample (2) are less than 1. NC, no template control. (TIF) [file pone.0053325.s002.tif]
