# Supplementary material for: Polymeric LabChip Real-Time PCR as a Point-of-Care-Potential Diagnostic Tool for Rapid Detection of Influenza A/H1N1 Virus in Human Clinical Specimens
Source: PLoS One. 2012 Dec 28;7(12):e53325. doi: 10.1371/journal.pone.0053325 (PMC3532060; doi:10.1371/journal.pone.0053325)
Supplement: Table S1 — Comparison of Ct values from tube-type and LabChip real-time PCR. (DOC) [file pone.0053325.s003.doc]

**Table S1.** Comparison of Ct values from tube-type and LabChip real-time PCR.

| **Clinical sample**  **No.** | **Tube-type PCR**  **(Bio-Rad CFX96)** | **LabChip PCR**  **(NanoBioSys G2-3)** | **Clinical sample**  **No.** | **Tube-type PCR**  **(Bio-Rad CFX96)** | **LabChip PCR**  **(NanoBioSys G2-3)** |  |
| --- | --- | --- | --- | --- | --- | --- |
| **2** | 28.03 | 27.38 | **127** | 24.40 | 29.20 | |
| **3** | 23.21 | 25.25 | **128** | 26.95 | 29.00 | |
| **4** | 28.13 | 22.33 | **132** | 25.36 | 28.67 | |
| **9** | 28.01 | 20.75 | **136** | 23.07 | 21.40 | |
| **13** | 23.20 | 24.67 | **138** | 22.96 | 30.00 | |
| **13** | 26.09 | 21.17 | **140** | 25.74 | 24.00 | |
| **14** | 21.62 | 25.10 | **141** | 24.08 | 23.17 | |
| **17** | 23.22 | 22.00 | **144** | 26.68 | 25.00 | |
| **18** | 22.97 | 19.75 | **145-1** | 27.20 | 26.67 | |
| **22** | 27.53 | 26.67 | **145-2** | 21.91 | 20.00 | |
| **23** | 28.92 | 27.63 | **149-1** | 23.67 | 29.86 | |
| **47** | 23.96 | 30.00 | **149-2** | 28.96 | 28.80 | |
| **51** | 24.26 | 26.67 | **152-1** | 23.32 | 24.00 | |
| **60** | 23.22 | 27.86 | **152-2** | 28.17 | 30.00 | |
| **68** | 20.34 | 19.50 | **155** | 22.42 | 21.25 | |
| **76** | 22.15 | 19.33 | **156** | 28.04 | 28.83 | |
| **78-1** | 22.65 | 20.00 | **157** | 27.26 | 23.33 | |
| **78-2** | 20.24 | 19.50 | **161** | 25.02 | 21.20 | |
| **81** | 23.48 | 20.60 | **168** | 24.14 | 20.00 | |
| **88** | 23.23 | 24.57 | **170** | 25.54 | 26.60 | |
| **89-1** | 22.82 | 24.00 | **173** | 24.21 | 28.06 | |
| **89-2** | 22.29 | 21.00 | **175** | 24.02 | 26.00 | |
| **93** | 27.24 | 26.22 | **183** | 28.97 | 30.00 | |
| **95** | 26.30 | 20.33 | **184** | 28.61 | 24.75 | |
| **96** | 27.37 | 27.60 | **185** | 24.06 | 19.57 | |
| **97** | 20.63 | 30.00 | **193** | 22.02 | 26.67 | |
| **98** | 23.71 | 30.00 | **200** | 29.42 | 30.00 | |
| **99** | 27.63 | 29.80 | **202-1** | 23.68 | 25.29 | |
| **102** | 27.41 | 27.00 | **202-2** | 25.45 | 30.00 | |
| **104** | 24.33 | 23.8 | **209** | 27.58 | 22.60 | |
| **106** | 21.06 | 22.14 | **210** | 24.96 | 26.60 | |
| **109** | 23.58 | 27.33 | **213** | 25.08 | 26.67 | |
| **114** | 25.09 | 21.67 | **215** | 24.52 | 25.22 | |
| **119** | 24.01 | 27.86 | **216** | 26.06 | 18.17 | |
| **120** | 27.88 | 20.50 | **225** | 26.24 | 23.44 | |
| **124** | 23.42 | 21.67 | **227** | 22.35 | 27.67 | |
